# *In vitro* and *in vivo* antimicrobial activity of protoberberine alkaloids as novel therapeutic candidates against *Mycoplasma hyopneumoniae*

**DOI:** 10.1128/spectrum.03254-25

**Published:** 2026-01-23

**Authors:** Chenchen Wang, Xiaoxu He, Lijun Yang, Yulin Qian, Xiaodan Li, Xuecheng Duan, Huifang Ma, Zhaoran Zhang, Xiangru Wang, Chen Tan

**Affiliations:** 1 National Key Laboratory of Agricultural Microbiology, College of Veterinary Medicine, Huazhong Agricultural University 627716, Wuhan, Hubei, China; 2 Hubei Hongshan Laboratory, Wuhan, Hubei, China; 3 Frontiers Science Center for Animal Breeding and Sustainable Production, Wuhan, Hubei, China; 4 Key Laboratory of Preventive Veterinary Medicine in Hubei Province, Wuhan, Hubei, China; 5 The Cooperative Innovation Center for Sustainable Pig Production, Wuhan, Hubei, China; Seton Hall University, South Orange, New Jersey, USA

**Keywords:** antibacterial, *M. hyopneumoniae*, protoberberine alkaloids, jatrorrhizine, treatment

## Abstract

*Mycoplasma hyopneumoniae (M. hyopneumoniae,* Mhp), the primary causative agent of swine enzootic pneumonia, poses significant threats to the pork industry, challenging food safety and sustainable development of the livestock industry. Here, four protoberberine alkaloids—epiberberine, jatrorrhizine, berberine, and coptisine—were identified by screening multiple natural compounds, and their anti-Mhp activity was evaluated. All alkaloids exhibited potent inhibitory effects against the virulent Mhp strain ES-2, with minimum inhibitory concentrations from 8 to 32 µg/mL. Their bactericidal activities were time- and concentration-dependent. At the cellular level, all protoberberine alkaloids significantly enhanced infected cell viability, suppressed pro-inflammatory cytokine expression (TNF-α, IL-6, and IL-1β), and exhibited low cytotoxicity toward host cells. In a Chang Da binary cross-breeding pig infection model, jatrorrhizine markedly alleviated clinical symptoms, reduced pulmonary pathogen loads, and mitigated histopathological damage in lung tissues, with therapeutic efficacy comparable to that of florfenicol. Our findings demonstrated that protoberberine alkaloids possessed potent anti-Mhp activity, high safety profiles, and promising therapeutic potential.

**IMPORTANCE:**

Swine enzootic pneumonia, caused by *Mycoplasma hyopneumoniae* (*M. hyopneumoniae*), remains one of the most economically devastating respiratory diseases in the global swine industry. The emergence of antibiotic resistance in livestock highlights the urgent need for effective, safe, and sustainable alternatives. This study demonstrates that naturally derived protoberberine alkaloids exhibit potent antibacterial activity against *M. hyopneumoniae* while maintaining low host cytotoxicity and strong anti-inflammatory effects. Among them, jatrorrhizine showed remarkable therapeutic efficacy in infected pigs, comparable with that of florfenicol. These findings provide a scientific basis for developing protoberberine alkaloids as promising natural alternatives to conventional antibiotics for controlling *M. hyopneumoniae* infections, thereby contributing to improved animal health, reduced antimicrobial resistance, and sustainable swine production.

## INTRODUCTION

The swine industry is a vital component of animal husbandry (1). In China, pork is not only the primary source of animal protein in the human diet but also a cornerstone of national food security and social stability (2). According to data from the Food and Agriculture Organization of the United Nations (FAO), Chinese pork production reached 57.94 million tons by 2023, accounting for 60% of the national meat output (3). Pork consumption holds a central place in the dietary structure of the Chinese population, with the highest per capita consumption (4). Therefore, maintaining a healthy and stable swine production system is crucial for ensuring pork supply and promoting sustainable agricultural development. However, with the expansion of intensive farming, swine herds are increasingly threatened by various infectious diseases. Respiratory and systemic diseases caused by bacterial pathogens are major factors that affect pig health and production efficiency (1, 5, 6). Among these pathogens, *Mycoplasma hyopneumoniae* (*M. hyopneumoniae*, Mhp), the primary etiological agent of enzootic pneumonia, is globally prevalent and is a core pathogen within the porcine respiratory disease complex (7, 8). The average prevalence of Mhp in domestic pig populations worldwide has been reported to be 30%–80% (9). Some studies indicated that Mhp infection could reduce the growth rate by 6%–16%, decrease the feed conversion ratio, prolong the fattening cycle, and increase mortality in severe cases (10–12). Moreover, Mhp infection heightens susceptibility to porcine reproductive and respiratory syndrome virus (PRRSV), swine influenza virus, and *Actinobacillus pleuropneumoniae*, aggravating lung pathology and economic loss (13, 14). In addition, chronic Mhp infection compromises muscle development and meat quality, lowering carcass grades and economic value (15, 16). Given the significant impact of Mhp, the development of effective prevention and control strategies has become a focal point in swine health research.

Currently, the control of Mhp infection relies on a combination of vaccination, environmental management, and pharmacological interventions. Among these, vaccination is widely implemented on large-scale pig farms to reduce clinical symptoms, lung lesions, and the incidence of secondary infections (17). Studies have shown that commercially available inactivated vaccines can enhance the host’s immune defense to some extent. However, owing to the high antigenic variability of the pathogen and the limited ability of the vaccine to induce mucosal immunity, protection remains suboptimal (15, 17, 18). Additionally, early post-weaning immunization is required, and improper management or inconsistent immune status across piglets can lead to vaccine failure (19). Considering the limited efficacy of vaccines, antibiotic therapy remains a key approach for clinical management of Mhp infections. Common antibiotics include macrolides (e.g., tilmicosin and tylosin), lincosamides (e.g., lincomycin and clindamycin), tetracyclines, and fluoroquinolones (20). Among these, tilmicosin and florfenicol (FFC) are frequently used because of their high tissue penetration and accumulation in lung tissue. These antibiotics are often administered via feed or drinking water to facilitate mass administration (21, 22). However, the widespread and often irrational use of antibiotics has raised significant concerns. As a cell wall-deficient gram-negative-like organism, Mhp is intrinsically resistant to β-lactam antibiotics, and increasing resistance to traditionally effective antibiotics has been reported in recent years (17, 20). Several studies have documented increasing minimum inhibitory concentrations (MICs) for tylosin, lincomycin, doxycycline, and other commonly used drugs in clinical isolates (23, 24). Given that Mhp may act as a reservoir for resistance genes, there is the potential for horizontal gene transfer to other bacteria, posing a public health threat (15). Consequently, there is an urgent need to develop novel antimicrobial agents that are effective against Mhp.

In recent years, natural products, particularly monomeric compounds derived from traditional Chinese medicines, have garnered attention as promising candidates for novel antibiotic development because of their unique structures, diverse bioactivities, and abundant sources (25, 26). However, compared with model pathogens such as *Staphylococcus aureus* or *Escherichia coli*, research on the antimicrobial potential of natural products against Mhp remains limited (27). Given the unique biological characteristics of Mhp, including its lack of a cell wall, metabolic dependence, and distinctive drug targets, it is likely to exhibit a susceptibility profile distinct from that of other bacteria (28), highlighting the need for targeted screening and evaluation of natural compounds. The virulent, drug-resistant Mhp ES-2 isolate (Hubei, China, 2017), characterized by typical mycoplasmal morphology and reproducible pneumonia in pigs (29), was selected for this study. A total of 20 structurally diverse natural compounds, including flavonoids, alkaloids, and phenolics, were screened based on their known safety profiles and pharmacological activities (30, 31). Among these compounds, protoberberine alkaloids, including epiberberine, jatrorrhizine, berberine, and coptisine, were selected for further investigation due to their antibacterial activity against Mhp. Previous studies have shown that berberine could inhibit the growth and adhesion of avian *Mycoplasma synoviae* in macrophages and attenuate PI3K/Akt-dependent inflammatory and apoptotic responses, suggesting its therapeutic potential in mycoplasma infections (32). In addition, several traditional herbal formulations containing berberine or matrine (e.g., Maxing Shigan decoction and Dang-Shen-Yu-Xing decoction) have been reported to improve clinical or experimental outcomes in children or calves infected with *Mycoplasma pneumoniae* (33, 34). However, these effects are largely attributed to the modulation of host immune responses, and direct evidence regarding the anti-mycoplasmal activity of individual monomer compounds remains limited. Beyond berberine, other protoberberine alkaloids such as epiberberine, jatrorrhizine, and coptisine also exhibit diverse antibacterial and anti-inflammatory activities (35–37). Nonetheless, previous research has predominantly focused on non-mycoplasmal pathogens or non-respiratory diseases, and whether these alkaloids display activity against Mhp remains poorly understood. To date, no systematic investigation has compared the efficacy of multiple protoberberine monomers against Mhp, nor has their *in vivo* performance been evaluated. Therefore, this study systematically assessed the anti-Mhp activities of four protoberberine alkaloids and conducted cellular assays and animal infection experiments to explore their translational potential. In particular, jatrorrhizine was highlighted as a key candidate, with the aim of determining whether it could serve as a promising therapeutic molecule against porcine mycoplasmosis, thereby providing experimental evidence for the applicability of protoberberine alkaloids in controlling Mhp infection.

## RESULTS

### Protoberberine alkaloids exhibited inhibitory potential against Mhp

**Fig 1:**
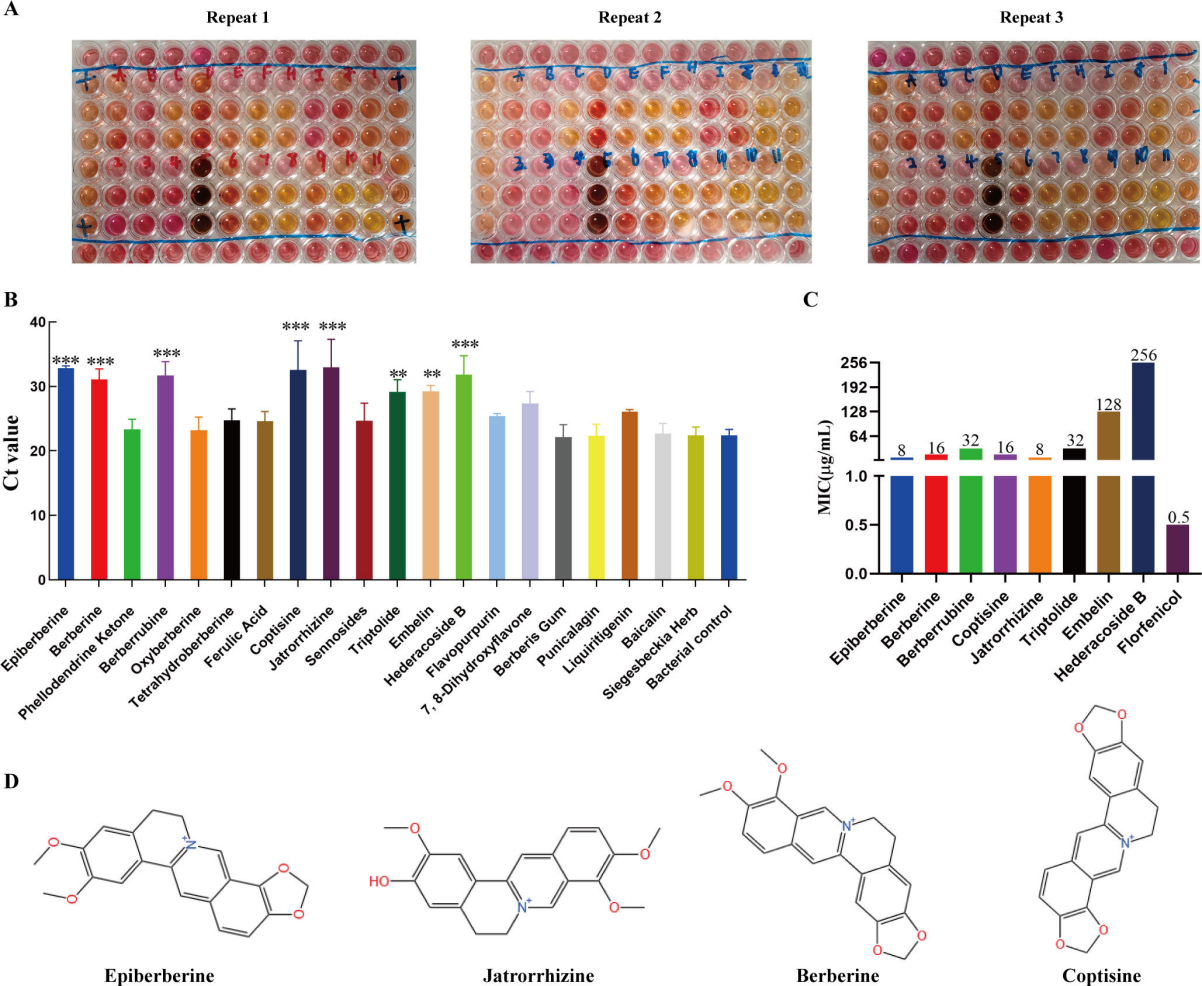
Screening of natural compounds for anti-Mhp activity. (**A**) Preliminary screening images of natural compounds at a concentration of 128 µg/mL using a 96-well culture plate system with the ES-2 strain. Experiments were repeated in triplicate. (**B**) Quantitative polymerase chain reaction-based detection of Mhp genomic DNA using Ct values to assess bacterial load, where a higher Ct value indicated lower bacterial DNA content and stronger antibacterial effect. (**C**) MICs of eight candidate compounds and florfenicol determined by broth microdilution. (**D**) Chemical structures of four final candidate compounds, which are all protoberberine alkaloids: epiberberine, jatrorrhizine, berberine, and coptisine. Data were mean values ± standard deviation of at least three biological replicates (*N* = 3). **P < 0.01, ***P < 0.001.

To identify natural compounds with significant *in vitro* antibacterial activity against Mhp strain ES-2, we screened 20 structurally diverse natural products with well-established safety profiles and pharmacological activities. These compounds were selected based on their structural diversity and natural origins. The compound layout in the 96-well plate is shown in Table S1. Based on visual inspection of the culture color changes (yellowing) from at least three independent replicates, several treatment groups, including epiberberine (Epi), jatrorrhizine (Jatr), berberine (BBR), and coptisine (Copt), exhibited no color change, suggesting potential inhibitory effects on Mhp growth (Fig. 1A). In contrast, several other groups showed similar color changes as the positive control, indicating a lack of apparent antibacterial activity (Fig. 1A). To quantitatively evaluate the antibacterial efficacy of each compound, Mhp genomic DNA was extracted from each treatment group, and the Mhp-specific gene P36 was detected using conventional quantitative polymerase chain reaction (qPCR). Changes in Ct values were used to assess the abundance of target DNA, with increased Ct values, indicating reduced bacterial DNA load and thus stronger antibacterial effects. As shown in Fig. 1B, compared with that of the positive control (Ct = 22.40), Ct values of Epi (32.83), BBR (31.07), berberrubine (31.68), Copt (32.55), Jatr (32.95), and hederacoside B (31.86) were significantly elevated (*P* < 0.001), indicating marked suppression of Mhp growth. In addition, triptolide and emodin showed moderate inhibitory activities (*P* < 0.01) (Fig. 1C). Other compounds, including flavonoids and phenolics (e.g., liquiritin, baicalin, and ferulic acid), did not significantly alter the Ct values, suggesting weak or negligible activity (Fig. 1C). Based on the qPCR screening results, eight compounds with significantly increased Ct values were selected for further evaluation of their MICs using the broth microdilution method. Among them, Epi and Jatr exhibited the strongest activity, with MICs of 8 µg/mL, followed by BBR and Copt (MIC = 16 µg/mL). Berberrubine and triptolide showed moderate activity (MIC = 32 µg/mL), and embelin and hederacoside B were the least effective (MICs of 128 and 256 µg/mL, respectively). As a positive control, FFC exhibited the lowest MIC (0.5 µg/mL); however, due to concerns over its side effects and resistance, safer natural compounds remain of interest for therapeutic development. Finally, the four most potent compounds, Epi, Jatr, BBR, and Copt, were selected for further antibacterial investigation. The chemical structures are shown in Fig. 1D. All four are protoberberine alkaloids characterized by a common polycyclic aromatic scaffold with multiple methoxy or hydroxy substituents, suggesting that such structural features may be critical to their antimicrobial activity. In summary, we successfully identified four protoberberine alkaloids with potent *in vitro* activity against Mhp ES-2, which were selected from a structurally diverse panel of natural products.

### Four protoberberine alkaloids exhibited effective bactericidal activity against Mhp

**Fig 2:**
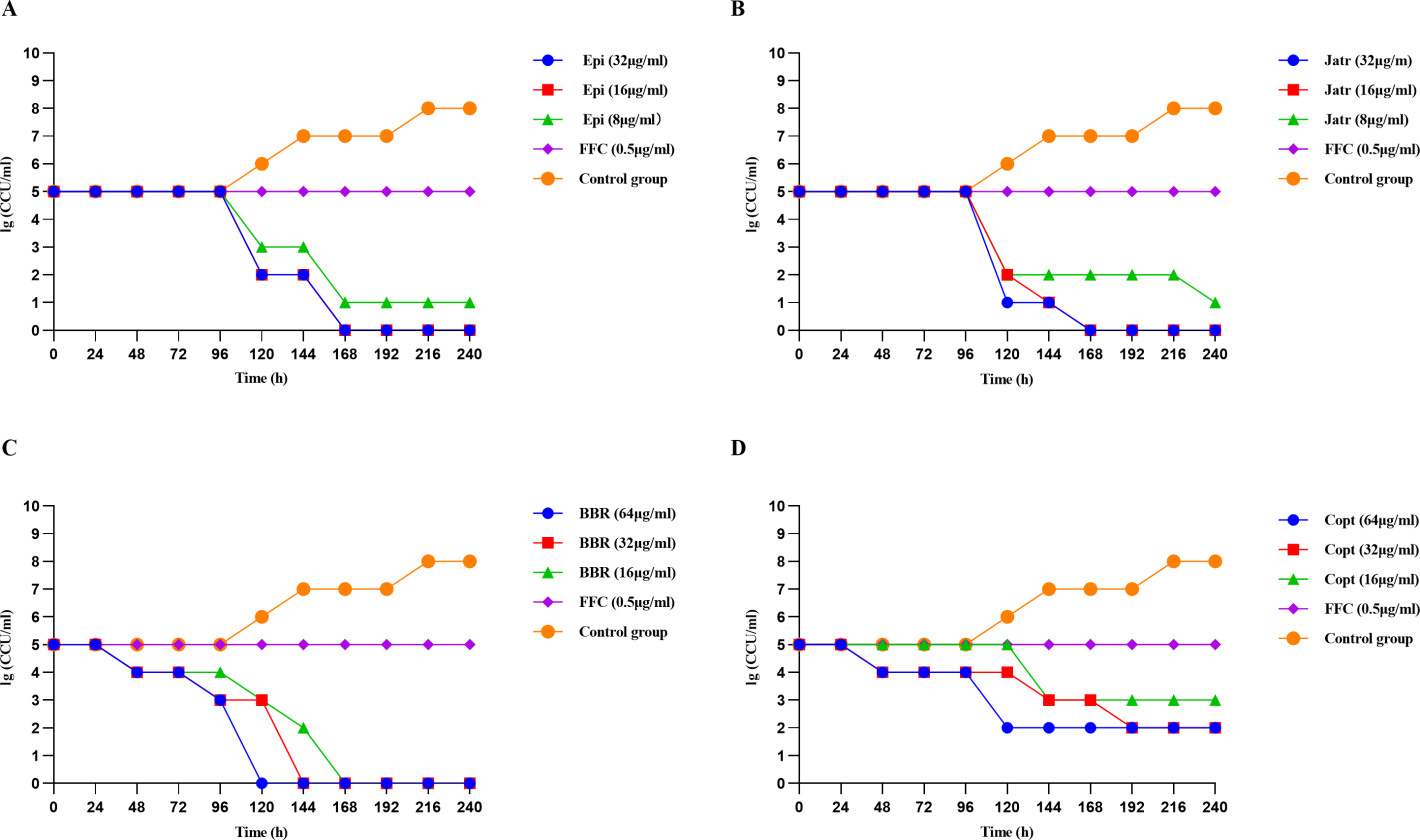
Time-kill kinetics of four protoberberine alkaloids against Mhp ES-2. Bacterial viability was assessed via lg(color-changing unit [CCU]/mL) over time after treatment with different concentrations of (**A**) Epi, (**B**) Jatr, (**C**) BBR, and (**D**) Copt. Each compound was tested at three concentrations, with florfenicol (FFC, 0.5 µg/mL) as a positive control and an untreated group as the control group. Sampling was performed every 24 h from 0 to 240 h, and CCU values were used to evaluate viable bacterial counts. Data were mean values ± standard deviation of three biological replicates (*N* = 3). Bactericidal activity was defined as ≥3 log10 reduction in viable count relative to baseline or counts below the LOD.

To validate the *in vitro* antibacterial activity of the four selected protoberberine alkaloids against the virulent Mhp ES-2 strain, a range of concentrations was tested, and bacterial viability was continuously monitored using the color-changing unit (CCU) method. FFC (0.5 µg/mL) and an untreated bacterial control were included for comparison. Overall, all four alkaloids exhibited significant bactericidal activity at higher concentrations (≥ 32 µg/mL), achieving noticeable or complete bacterial clearance within 96‒168 h (Fig. 2). Notably, at a concentration of 32 µg/mL, both Epi and Jatr achieved complete eradication within 168 h, with highly similar kill curves, suggesting comparable efficacy (Fig. 2A and B). At a concentration of 64 µg/mL, BBR showed the most rapid bactericidal effect, with bacterial counts steadily decreasing from 96 h and decreasing to below the detection limit within 120 h, indicating its more rapid effect (Fig. 2C). In contrast, Copt exhibited weaker activity, with incomplete killing even at a concentration of 64 µg/mL, suggesting lower potency (Fig. 2D). A clear dose‒response relationship was observed for all compounds; increased concentrations led to reduced bacterial survival, and higher doses resulted in more rapid and more complete bactericidal effects (Fig. 2). Despite their structural similarities to protoberberine alkaloids, the four compounds showed varying levels of *in vitro* activity against Mhp, implying that subtle differences in substitution patterns, spatial conformation, and membrane permeability may have influenced their antibacterial performance. The rapid action of BBR may have been attributed to its high lipophilicity and efficient target-binding abilities. In summary, all four protoberberine alkaloids effectively inhibited Mhp ES-2 growth *in vitro*, with strong time- and concentration-dependent antibacterial activities. BBR and Jatr were identified as the most effective treatments.

### Synergistic antibacterial activity among four protoberberine alkaloids

To further investigate potential synergistic antibacterial effects of different protoberberine alkaloids, a checkerboard microdilution assay was performed to assess their combined antibacterial activities in *vitro*. As shown in Table 1, a combination of Epi and the other three alkaloids exhibited additive effects. Specifically, the fractional inhibitory concentration index (FICI) for Epi combined with Jatr (Epi + Jatr) was 0.75, that with BBR (Epi + BBR) was 1.0, and that with Copt (Epi + Copt) was 0.5625. The combination of BBR + Jatr also showed an additive effect, with an FICI of 0.625 (Table 1). These results suggested that the antibacterial activity of these combinations was enhanced compared to that with the use of individual compounds, although no synergistic effects were observed. However, the combinations of BBR + Copt and Copt + Jatr yielded FICI values of 1.5, indicating no significant interactions between these compound pairs (Table 1). When combined with the conventional antibiotic FFC, all tested combinations showed FICI values > 1.0, indicating neither additive nor synergistic effects (Table 1). In summary, certain combinations of protoberberine alkaloids, particularly Epi + Jatr, Epi + Copt, and BBR + Jatr, demonstrated potential additive antibacterial effects against Mhp, whereas co-treatment with FFC did not enhance antibacterial efficacy. These findings suggested that protoberberine alkaloids are better suited as stand-alone natural antibacterial agents.

**TABLE 1:** Combination of drugs with antibacterial results^*a*^

Combination group	FICI	Judgment
Epi + Jatr	0.75	Additivity
Epi + BBR	1.00	Additivity
Epi + Copt	0.56	Additivity
BBR + Copt	1.50	Indifference
BBR + Jatr	0.63	Additivity
Copt + Jatr	1.50	Indifference
Epi + FFC	2.50	Indifference
BBR + FFC	1.50	Indifference
Copt + FFC	3.00	Indifference
Jatr + FFC	4.00	Indifference

^*a*^ Epi, Epiberberine; BBR, Berberine; Jatr, Jatrorrhizine; and FFC, Florfenicol.

### Limited cytotoxicity of protoberberine alkaloids in mammalian cells

**Fig 3:**
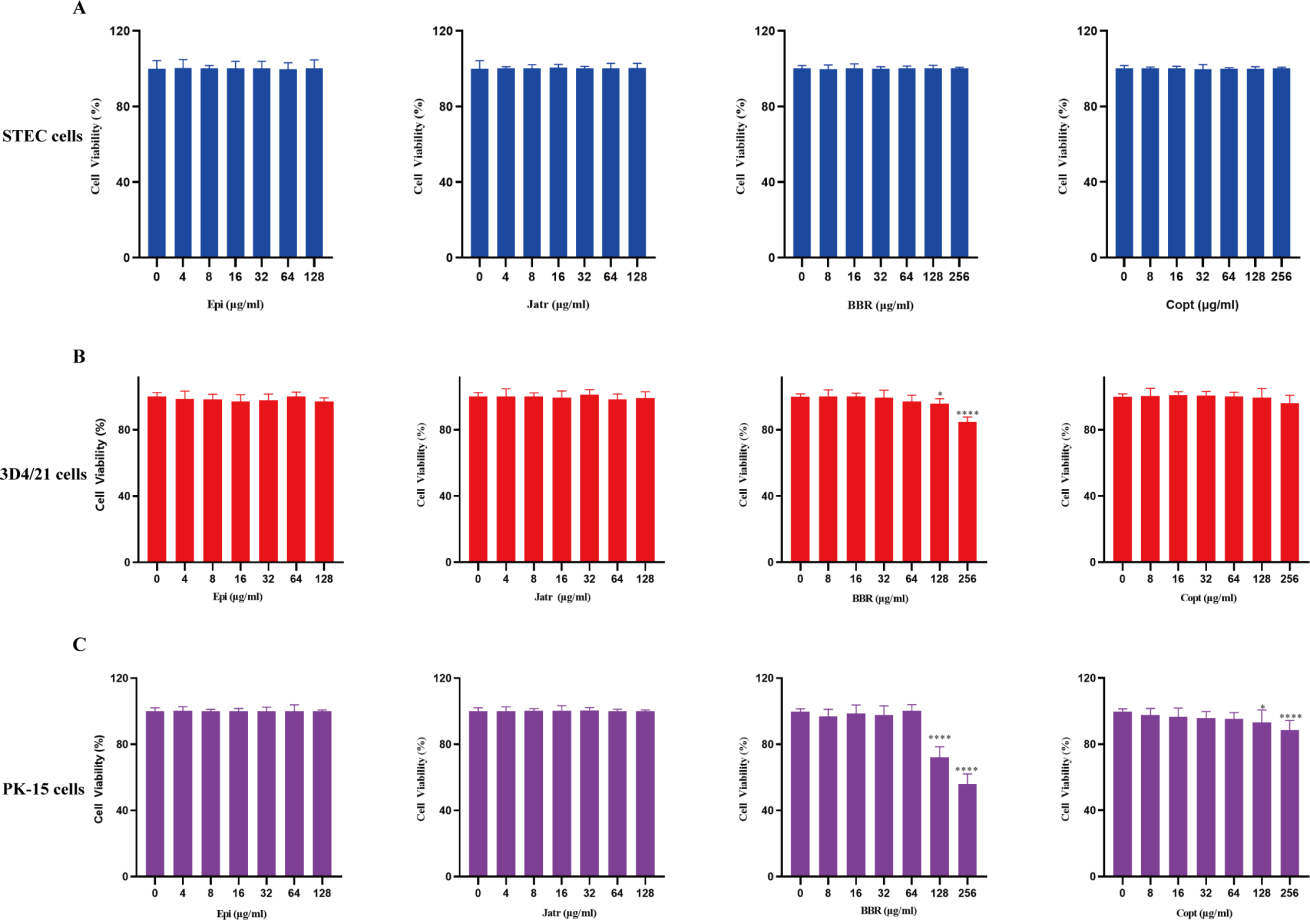
Cytotoxic effects of the four protoberberine alkaloids at different concentrations in (**A**) STEC, (**B**) porcine alveolar macrophages (3D4/21), and (**C**) porcine kidney epithelial cells (PK-15). Data were mean values ± standard deviation of at least three biological replicates (*N* = 9). *P < 0.05, ****P < 0.0001.

Natural alkaloids have garnered interest as novel antimicrobial candidates because of their broad pharmacological activities and natural origins. However, their safety profiles are critical to their clinical translation. In this study, the cytotoxicity of four protoberberine alkaloids was systematically evaluated in three representative porcine-derived cell models: swine tracheal epithelial cells (STEC), porcine kidney epithelial cells (PK-15), and porcine alveolar macrophage cells (3D4/21). STEC cells derived from the porcine tracheal epithelium are an ideal model for assessing airway-localized irritation and barrier toxicity. The 3D4/21 cell line represents alveolar macrophages and is critical for evaluating pulmonary immunotoxicity and drug-targeted safety. The PK-15 cell line, which is derived from the kidney epithelium, is a classical model for systemic and renal toxicity screening. This combination of cell lines enables a comprehensive assessment of the biocompatibility and safety of compounds across organ systems. In STEC cells, both Epi and Jatr exhibited negligible cytotoxicity within the 4‒128 µg/mL concentration range, maintaining cell viability > 99% (Fig. 3A). Similarly, BBR and Copt were non-toxic in the 8‒256 µg/mL range, with stable cell viability around 99%, suggesting high biosafety in airway epithelial cells (Fig. 3A). In 3D4/21 alveolar macrophages, Epi, Jatr, and Copt showed minimal cytotoxicity at all tested concentrations, with cell viability remaining at 99% (Fig. 3B). In contrast, BBR exhibited slight cytotoxicity at concentrations > 128 µg/mL, with viability decreasing to 95%, indicating the need for caution at higher doses (Fig. 3B). In PK-15 cells, Epi and Jatr again showed no detectable cytotoxicity at concentrations ≤ 128 µg/mL (Fig. 3C). However, BBR exhibited significant toxicity at concentrations > 128 µg/mL, reducing cell viability to 72% (Fig. 3C). Copt also induced mild toxicity, decreasing cell viability to 93% (Fig. 3C). These results highlight the potential renal toxicity risks of high BBR and Copt concentrations. In contrast, the clinical antibiotic FFC exhibited minimal cytotoxicity in all three cell lines within the tested concentration ranges (Fig. S1). Collectively, these findings suggested that Epi and Jatr exhibited favorable biocompatibility in porcine respiratory and renal epithelial cell lines, whereas BBR and Copt require dose optimization to ensure their safety, particularly in kidney-derived cells.

### Protective effects of protoberberine alkaloids against Mhp*-*infected cells

**Fig 4:**
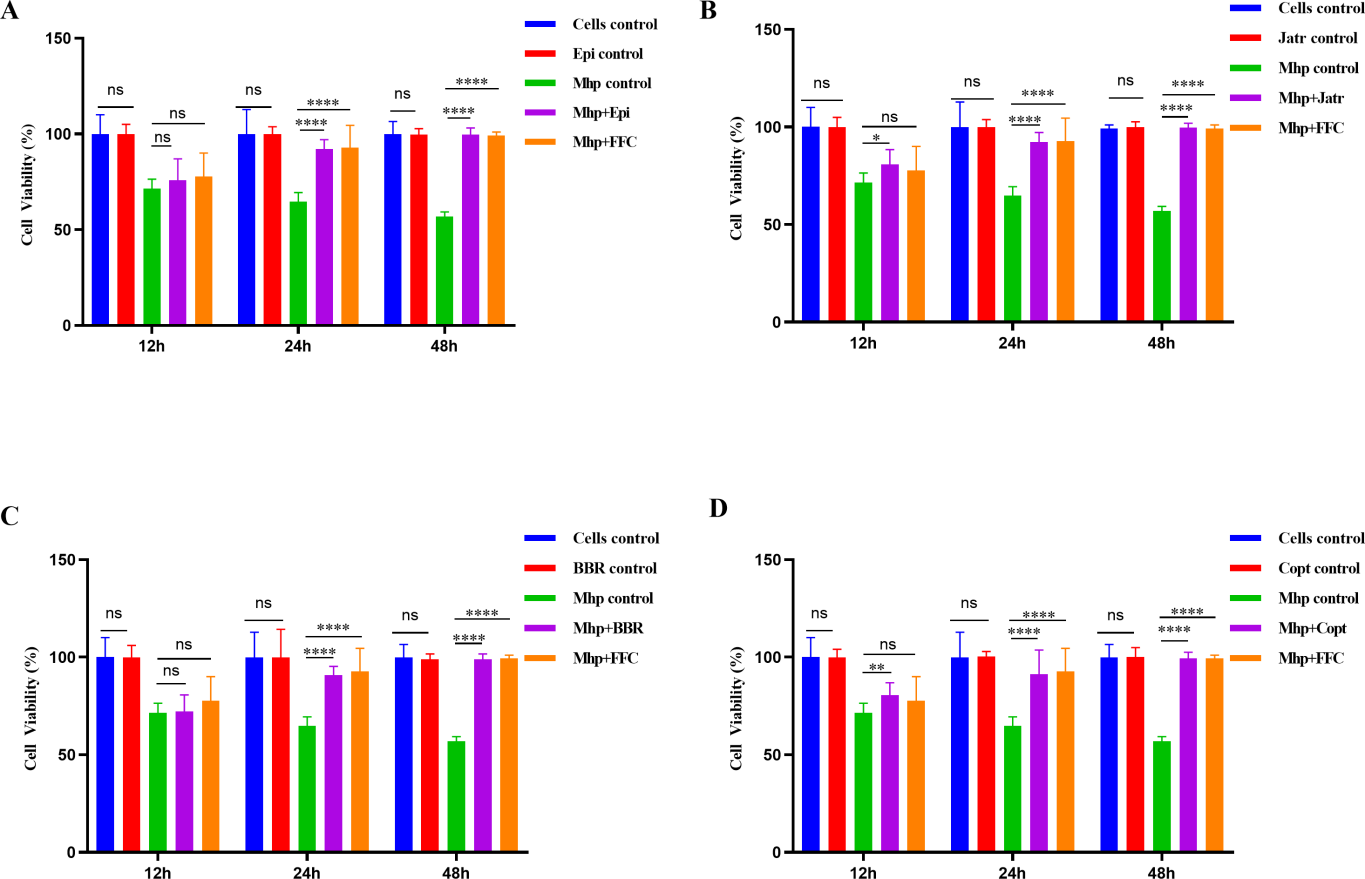
Temporal changes in cell viability of Mhp ES-2-infected cells treated with (**A**) Epi, (**B**) Jatr, (**C**) BBR, and (**D**) Copt. Each compound was tested at its maximum non-cytotoxic concentration. Florfenicol (FFC) was used as a reference drug. Data were mean values ± standard deviation from three biological replicates (*N* = 25). ns, not significant, *P < 0.05, **P < 0.01, ****P < 0.0001.

To further evaluate the protective effects of the four protoberberine alkaloids against Mhp ES-2-infected cells, the highest non-toxic concentration of each compound was selected for treatment. Overall, the protoberberine alkaloids significantly improved cell viability at 24 and 48 h compared with that in the Mhp-infected group, indicating notable cytoprotective effects (Fig. 4). Particularly, at 48 h, cell viability in all treatment groups approached the levels observed in uninfected and FFC-treated control cells (Fig. 4). None of the protoberberine alkaloids alone (in the absence of infection) caused significant cytotoxicity compared with untreated control cells (*P* > 0.05), reaffirming their safety at the experimental concentrations (Fig. 4). Specifically, the Epi group showed a marked increase in cell viability from 75.99% at 12 h to 92.18% at 24 h, and viability was maintained at normal levels by 48 h (Fig. 4A). Similarly, the Jatr group showed an increase from 80.93% to 92.29% after 24 h (Fig. 4B). The BBR group demonstrated improved viability from 72.12% (12 h) to 90.92% (24 h) and sustained normal levels at 48 h (Fig. 4C). Viability of Copt-treated cells also increased from 80.7% at 12 h to 91.32% at 24 h, with no significant difference compared with that of the control at 48 h (Fig. 4D). These results confirmed that all four compounds effectively restored the viability of Mhp-infected cells, with Epi and Jatr showing particularly strong protective effects. This suggested that their protective role may have been attributed to inhibition of Mhp proliferation or enhancement of host cell repair mechanisms.

### Regulatory effects of four protoberberine alkaloids on inflammatory cytokine expression in Mhp-infected cells

**Fig 5:**
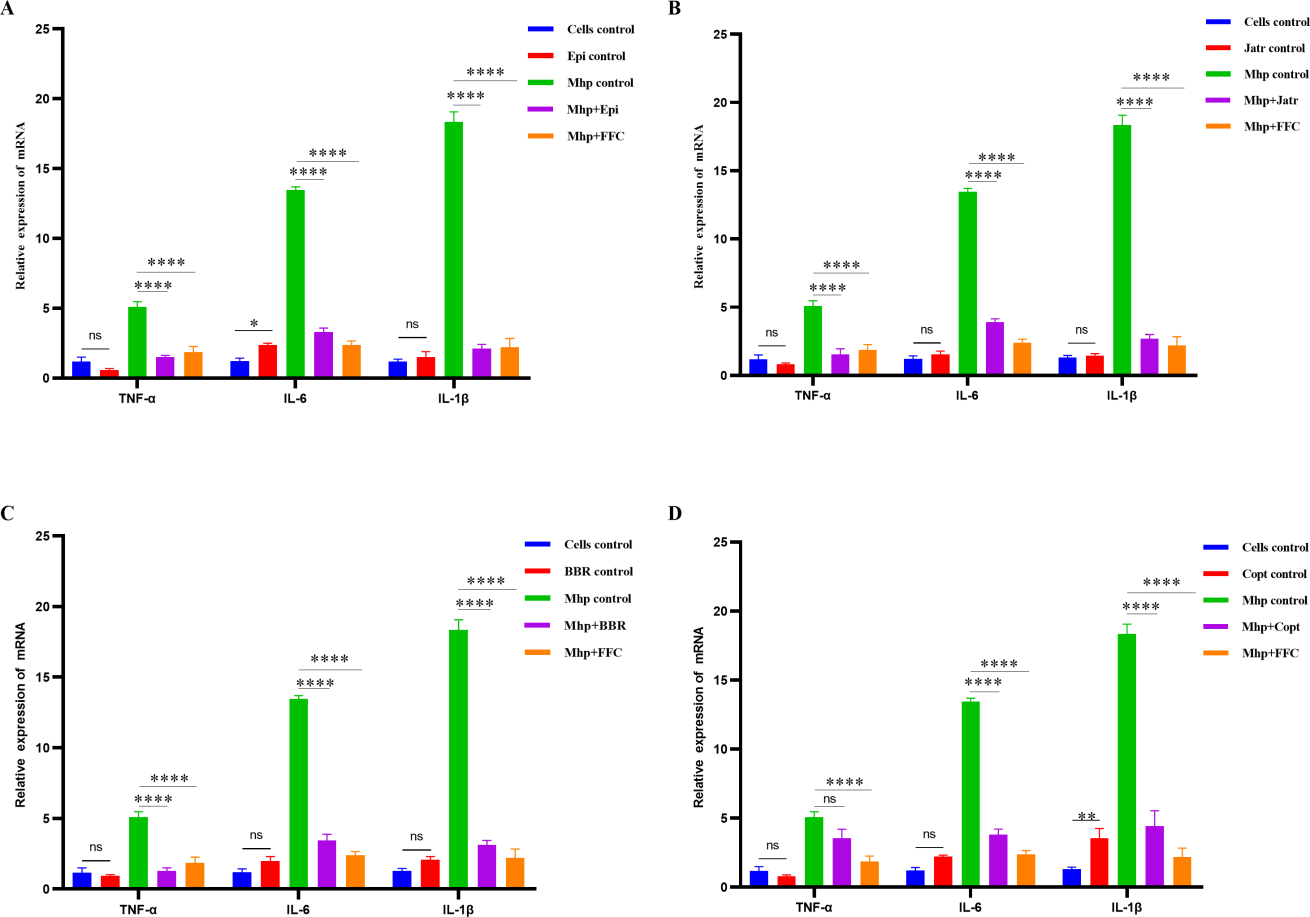
Effects of four alkaloids on pro-inflammatory cytokine expression in Mhp-infected cells. (**A**) Epi, (**B**) Jatr, (**C**) BBR, and (**D**) Copt were tested for their ability to regulate TNF-α, IL-6, and IL-1β mRNA levels in alveolar macrophages infected with Mhp. The experimental groups included untreated control cells, compound-only groups (uninfected), Mhp-infected control, Mhp + compound treatment, and Mhp + positive control (florfenicol [FFC]). Cytokine mRNA levels were measured via quantitative polymerase chain reaction using β-actin as a reference gene. Data were mean values ± standard deviation from at least three biological replicates. Statistical significance was determined by two-way analysis of variance: ns, not significant, *P < 0.05, **P < 0.01, ****P < 0.0001.

To further elucidate the modulatory effects of different alkaloids on the expression of inflammatory mediators induced by Mhp ES-2, mRNA levels of three representative pro-inflammatory cytokines, including TNF-α, IL-6, and IL-1β, were quantified in infected cells. The results showed that Mhp infection significantly upregulated the transcription of all three cytokines, with IL-6 and IL-1β expression increasing by nearly 15-fold and 20-fold, respectively, compared with that in the normal control group (Fig. 5). This indicated that Mhp infection strongly induced the release of inflammatory mediators. In contrast, treatment with all four alkaloids effectively reversed the infection-induced overexpression of inflammatory cytokines to varying degrees, with Epi and Jatr showing the most pronounced effects (Fig. 5). Notably, none of the alkaloids induced cytokine expression in uninfected cells, indicating that they did not elicit an inflammatory response on their own. Among the compounds, Epi exerted the strongest inhibitory effect, significantly suppressing TNF-α and IL-1β expressions to levels comparable with those achieved with the positive control drug FFC and restoring them to baseline levels (Fig. 5A). Although Epi did not completely normalize IL-6 expression, its level was markedly reduced compared with that in the infected group. Jatr also significantly inhibited TNF-α expression to normal levels and effectively downregulated IL-6 and IL-1β, demonstrating broad-spectrum anti-inflammatory activity (Fig. 5B). BBR showed a particularly strong suppressive effect on TNF-α and, similar to Jatr, significantly downregulated IL-1β and IL-6, albeit not to normal levels (Fig. 5C). Copt significantly suppressed IL-6 and IL-1β expressions but had minimal effect on TNF-α (Fig. 5D). These findings demonstrated that all four protoberberine alkaloids possessed anti-inflammatory activity to varying extents. In particular, Epi and Jatr were highly effective in suppressing multiple key inflammatory cytokines and mitigating inflammation triggered by Mhp-infected cells, thereby highlighting their potential as immunomodulatory agents for the treatment of Mhp-associated infections.

### Jatrorrhizine exhibited excellent therapeutic efficacy against porcine mycoplasmal pneumonia

**Fig 6:**
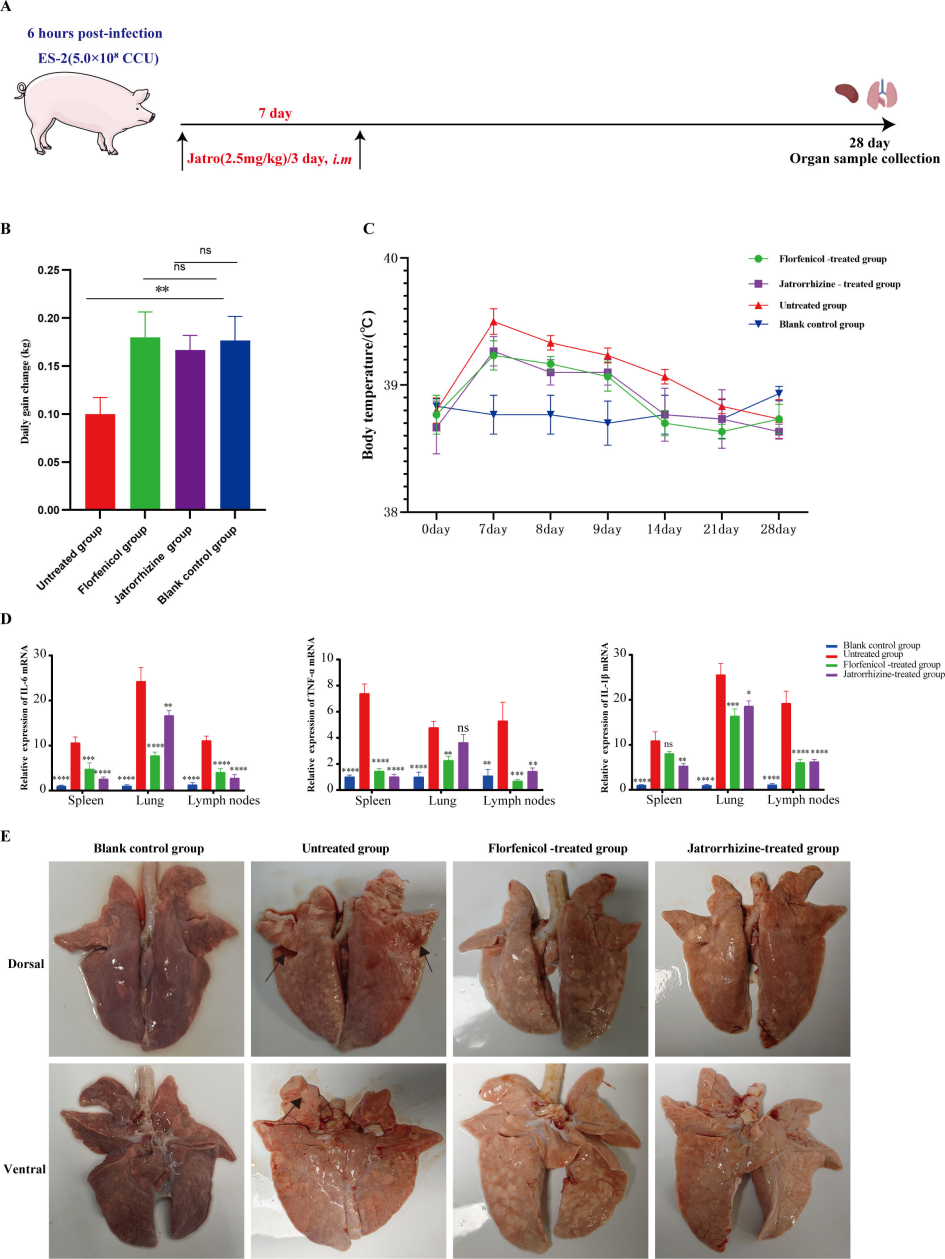
Evaluation of the therapeutic efficacy of jatrorrhizine in a swine model infected with Mhp ES-2. (**A**) Schematic of the *in vivo* experimental protocol: animals were administered jatrorrhizine (2.5 mg/kg, *i.m.*, three times in total) beginning 6 h post-infection and sacrificed on day 28 for tissue collection. (**B**) Changes in average daily weight gain across groups. (**C**) Temperature monitoring curves. (**D**) Relative mRNA expression of TNF-α, IL-6, and IL-1β in the spleen, lung, and hilar lymph nodes. (**E**) Gross morphology of lung tissues. Data were mean values ± standard deviation from at least three biological replicates. Statistical analysis was conducted using two-way analysis of variance. ns, not significant, *P < 0.05, **P < 0.01, ***P < 0.001, ****P < 0.0001.

Based on comprehensive *in vitro* antibacterial activity, cytotoxicity screening, and drug cost-effectiveness considerations, Jatr was selected as the most promising compound for further validation *in vivo* in a porcine infection model established using Mhp ES-2. The experimental protocol is illustrated in Fig. 6A. Body weight and temperature changes were monitored to assess clinical efficacy. Growth performance data revealed that the average daily gain (ADG) in the untreated infected group decreased to 0.10 kg, which was significantly lower than that observed in the uninfected control group (0.17 kg) (Fig. 6B). In contrast, the Jatr- and FFC-treated groups exhibited ADG values of 0.17 and 0.18 kg, respectively, which were both significantly higher than that of the infected group and statistically indistinguishable from those of healthy controls (Fig. 6B). This suggested that both treatments effectively restored growth performance. In terms of body temperature, pigs in the uninfected blank control group maintained normal ranges (38.6°C–39.0°C) (Fig. 6C). Following infection, untreated pigs exhibited temperature fluctuations ranging from 38.6°C to 39.6°C (Fig. 6C). However, Jatr- and FFC-treated animals returned to the physiological range (38.5°C‒39.4°C and 38.6°C‒39.3°C, respectively), indicating that both treatments were effective in resolving infection-induced pyrexia (Fig. 6C). Subsequently, tissue samples from the heart, liver, lungs, kidneys, trachea, and hilar lymph nodes were collected and subjected to qPCR analysis for pathogen detection. In the untreated group, all lung samples tested positive for Mhp (100% infection rate), and 33.33% of spleen and tracheal tissues also showed the presence of pathogens (Table 2). No Mhp DNA was detected in any of the tissues from the Jatr-treated, FFC-treated, or uninfected blank control groups, demonstrating that both treatments successfully eliminated the pathogen from host tissues (Table 2). In addition, the relative expression of TNF-α, IL-6, and IL-1β was assessed in the spleen, lung, and hilar lymph nodes. Compared with the untreated group, which showed significantly elevated cytokine levels, the Jatr-treated group exhibited a marked reduction in all three inflammatory markers (Fig. 6D). Gross and histopathological examinations were performed to evaluate lung tissue protection. Macroscopically, untreated infected lungs displayed significant lesions, particularly in the apical and cardiac lobes, with consolidated and meaty textures (Fig. 6E). No visible lesions were observed in the lungs of the uninfected control, Jatr-treated, or FFC-treated groups (Fig. 6E). Histologically, lung tissues from the untreated group showed characteristic features of bronchopneumonia and interstitial pneumonia with extensive inflammatory cell infiltration (Fig. S2). In contrast, the Jatr- and FFC-treated groups exhibited no significant histopathological changes, resembling the normal lung architecture observed in uninfected blank controls (Fig. S2). Taken together, these findings confirmed the reliability of the porcine Mhp infection model and demonstrated that Jatr significantly alleviated Mhp-induced systemic inflammation and lung tissue damage. This study provides strong experimental and theoretical support for the development of Jatr as a natural therapeutic candidate for the treatment of porcine mycoplasma pneumonia.

**TABLE 2:** Detection rate of *M. hyopneumoniae* in various tissues

Group	Heart	Liver	Spleen	Lung	Kidney	Trachea	Lymph nodes
	Detection rate of *M. hyopneumoniae* (%)						
Untreated group	0 (0/3)	0 (0/3)	33.33 (1/3)	100 (3/3)	0 (0/3)	33.33 (1/3)	0 (0/3)
Florfenicol-treated group	0 (0/3)	0 (0/3)	0 (0/3)	0 (0/3)	0 (0/3)	0 (0/3)	0 (0/3)
Jatrorrhizine-treated group	0 (0/3)	0 (0/3)	0 (0/3)	0 (0/3)	0 (0/3)	0 (0/3)	0 (0/3)
Blank control group	0 (0/3)	0 (0/3)	0 (0/3)	0 (0/3)	0 (0/3)	0 (0/3)	0 (0/3)

## DISCUSSION

Mhp infection not only severely compromises the respiratory function and production performance of pigs but also markedly affects carcass grading and muscle quality in market pigs, thereby reducing the sensory attributes and consumer acceptance of pork (17, 38). This represents a potential risk to food safety and undermines consumer confidence. Furthermore, current clinical treatments rely predominantly on antibiotics, which are increasingly challenged by increasing resistance rates. Excessive or improper use of antimicrobials also poses the risk of drug residues, threatening public health (39). Therefore, the development of natural antimicrobial alternatives with high efficacy, low toxicity, and minimal residue risk is of great significance for maintaining animal health, enhancing pork quality, ensuring food safety, and promoting sustainable and environment-friendly farming practices. In this study, we systematically evaluated the antimicrobial activity of four protoberberine alkaloids (Epi, Jatr, BBR, and Copt) against the clinical strain Mhp ES-2. Interestingly, all four alkaloids were obtained from the traditional medicinal plant *Coptis chinensis*. Our findings demonstrated multifaceted biological effects of these natural alkaloids in inhibiting Mhp proliferation, modulating inflammatory responses, and protecting against cellular injury, thereby highlighting their novel and practical potential. *In vitro* screening revealed that all four compounds strongly inhibited Mhp growth. Most studies on the effects of natural products against *Mycoplasma* have focused primarily on their immunomodulatory effects. Compounds such as baicalin, glycyrrhizin, and quercetin have been shown to alleviate *Mycoplasma*-induced inflammation via the inhibition of NF-κB or MAPK pathways (40–43). However, systematic evaluation of the direct antimicrobial activity of protoberberine alkaloids against *Mycoplasma*, particularly their MICs, remains limited. This study specifically addressed this knowledge gap by determining the MICs of the tested alkaloids against a virulent clinical Mhp strain, thereby providing critical evidence for their direct antimicrobial activity against *Mycoplasma*.

Time-kill kinetics revealed that the alkaloids exerted time- and dose-dependent bactericidal effects. Despite their shared structural class, differences in their bactericidal curves suggested that subtle variations in molecular substitution (e.g., methoxy groups and spatial conformations) significantly influenced their antibacterial activity (44). Previous structure-activity relationship studies have also indicated that methoxy substitution at specific positions can alter the binding affinity to cell membranes or target enzymes, thereby modulating the antimicrobial spectra and potency (44–46). Previous studies indicate that protoberberine alkaloids such as berberine act through multitarget antibacterial mechanisms (47). These include disruption of cell membrane architecture, interference with bacterial division via inhibition of FtsZ polymerization, and inhibition of fundamental metabolic processes of bacteria that limit pathogen survival (48–50). Although these mechanisms were established primarily in non-mycoplasmal bacteria, they offer reasonable biological hypotheses for how structurally related alkaloids might influence Mhp. Combination assays demonstrated the additive effects between Epi and Copt or Jatr and between BBR and Jatr (FICI values ranging from 0.56 to 0.75), although no synergism was observed. Nevertheless, these combinations may have enhanced the antimicrobial efficacy to a certain extent. In contrast, no additive or synergistic interactions were found between these alkaloids and conventional antibiotics, such as FFC, indicating that these compounds may be better suited for monotherapy or for use in combination with structurally similar natural products in phytochemical formulations. This approach could improve the antimicrobial activity while avoiding the metabolic interference often observed with antibiotic combinations.

In addition to their antimicrobial effects, all four alkaloids exhibited high biocompatibility and pronounced protective cellular-level effects. In porcine tracheal epithelial cells, alveolar macrophages, and renal epithelial cells, both Epi and Jatr showed no significant cytotoxicity at concentrations <128 µg/mL, with cell viability consistently >99%. In contrast, BBR demonstrated some cytotoxicity in PK-15 cells at concentrations >128 µg/mL, with viability decreasing to 72%, underscoring the importance of dose optimization for potential therapeutic applications. A previous study indicated that high BBR concentrations may induce cytotoxicity by disrupting the mitochondrial membrane potential or arresting the cell cycle (51, 52). Interestingly, at appropriate concentrations, all four alkaloids not only showed no cytotoxicity but also significantly improved cell viability in Mhp-infected models. Epi and Jatr restored cell viability to baseline levels after 48 h of treatment. These findings suggested that their protective effects may have arisen from both the inhibition of pathogen replication and the attenuation of inflammatory responses. This hypothesis is supported by the downregulation of key pro-inflammatory cytokines following treatment, with Epi and Jatr demonstrating particularly strong suppression of TNF-α and IL-1β, comparable with that of FFC. Thus, these compounds not only possessed direct antimicrobial activity but also mitigated the inflammatory response through modulation of inflammatory signaling, which is a dual mechanism rarely reported in anti-Mhp therapies.

*In vivo* validation demonstrated, for the first time, the therapeutic efficacy of Jatr in a swine Mhp infection model. A lower therapeutic dose significantly improved clinical parameters, such as body temperature, ADG, and pathogen clearance, with efficacy comparable with that of FFC. Moreover, Jatr reduced inflammatory cytokine levels, suggesting superior modulation of Mhp-associated immunopathology. Histopathological analysis confirmed a significant reduction in inflammation and pulmonary consolidation, with the tissue morphology resembling that of the uninfected control group. These results highlighted that Jatr controlled the pathogen load and preserved lung tissue integrity. Compared with previous studies on Jatr in rodent models of respiratory infection (53–55), this study provides therapeutic evidence in a large animal model, significantly enhancing its translational potential for novel drug development. A limitation of this study is the relatively small number of animals per group (*n* = 3). Although this sample size is in line with prior exploratory *in vivo* infection models and was determined in accordance with ethical considerations to minimize animal use, it does limit the statistical power of the analyses. The consistent trends observed across independent experiments lend support to the robustness of the findings; nevertheless, future studies with larger cohorts will be required to confirm these results and enable more definitive statistical comparisons. In addition, a key consideration for developing protoberberine alkaloids as antibiotic alternatives is whether prolonged exposure could induce resistance. Existing evidence suggests that berberine has a low probability of resistance development; *Escherichia coli* subjected to more than 200 passages in berberine-containing media exhibited no change in MIC, whereas the MICs of neomycin and cefotaxime increased by more than 10-fold under comparable conditions (56). This implies that berberine-like alkaloids may exert reduced selective pressure for resistance evolution. However, whether Mhp develops adaptive resistance during long-term exposure remains unknown. Therefore, systematic resistance evolution experiments will be needed to verify whether these compounds maintain efficacy over extended use in Mhp control. Overall, these protoberberine alkaloids demonstrated promising biological activity, safety, and tissue-protective properties, laying a solid foundation for their development as natural commercial anti-Mhp agents. Future work may incorporate transcriptomics and proteomics to elucidate the underlying molecular mechanisms, and large-scale field trials to evaluate real-world efficacy, thereby advancing novel antibiotic alternatives for controlling Mhp in swine farming.

### Conclusion

This study systematically characterized the antimicrobial potential of four protoberberine alkaloids against Mhp. These compounds not only demonstrated significant *in vitro* antibacterial and bactericidal activity but also effectively alleviated Mhp-induced cellular injury and inflammatory responses. Animal experiments confirmed the therapeutic efficacy of Jatr in alleviating clinical symptoms, eradicating pulmonary pathogens, and improving histopathological outcomes, with effects comparable with those of conventional antibiotics. Overall, protoberberine alkaloids exhibited multifunctional properties, including direct anti-Mhp activity, anti-inflammatory effects, and tissue protection, suggesting their potential as natural antimicrobial alternatives. This study provides a theoretical foundation and experimental evidence for the development of novel, low-toxicity, highly efficacious agents.

## MATERIALS AND METHODS

### Chinese herbal monomers, bacteria, cell lines, and culture conditions

Berberrubine, Epi, BBR, Copt, and FFC were purchased from Macklin Biochemical Co. Ltd. (Shanghai, China). All herbal monomers were prepared as 100 mg/mL stock solutions, sterilized using 0.22-µm filters, aliquoted into sterile 2-mL Eppendorf tubes, and stored at 4°C. FFC injection was obtained from Jiangxi New Century Animal Health Products Co., Ltd.. Mhp strain ES-2 was isolated from diseased pigs on a large-scale pig farm in Hubei Province, China. The strain was cultured in modified Friis medium and incubated at 37°C in a 5% CO₂ humidified atmosphere. STEC, 3D4/21, and PK-15 were maintained in our laboratory. All cell lines were maintained in dulbecco's modified eagle medium (DMEM) or Roswell Park Memorial Institute (RPMI)-1640 medium supplemented with 10% fetal bovine serum (FBS) and 1% penicillin‒streptomycin solution, and incubated at 37°C under 5% CO₂.

### Screening of herbal monomers

Twenty herbal monomers were diluted with Friis medium to a final concentration of 128 µg/mL and filtered through 0.22-µm membranes for sterilization. In a 96-well plate, rows 1 and 8 were filled with 200 µL of sterile medium as blank controls. Columns 2–11 and rows 2 and 7 served as drug-only controls (100 µL of medium + 100 µL of drug solution). Wells from columns 2–11 (except controls) were inoculated with 100 µL of Mhp (1 × 10⁵ CCU/mL) and 100 µL of herbal solution. Each compound was tested in quadruplicate, and experiments were performed independently in triplicate. The plates were sealed, wrapped in foil, and incubated at 37°C under 5% CO₂ until a color change occurred in control wells. Compounds in wells that showed no color change were identified as preliminary hits. Culture supernatants were collected, and total nucleic acids were extracted for qPCR (57, 58). The following cycling conditions were used: pre-denaturation at 95°C for 5 min, followed by 45 cycles at 95°C for 10 s and 60°C for 30 s. Primer sequences are listed in Table S2.

### MIC determination

This assay referred to the methods available in a previous study and optimized them appropriately (59). MICs of the selected herbal monomers against Mhp were determined using a modified broth microdilution method based on published protocols. The bacterial cultures were adjusted to 1 × 10 CCU/mL. Each well received 100 µL of bacterial suspension, and 100 µL of serially diluted drug solution (2-fold dilutions across 10 wells, starting from the maximum working concentration). Column 12 served as a positive control (bacteria only), and rows 1 and 8 served as negative controls (medium only). The plates were then incubated at 37°C for 10‒14 days. Color change in the phenol red indicator (red to yellow or orange) was used to assess growth. The MIC was defined as the lowest drug concentration that completely inhibited the color change. Each assay was performed in triplicate.

### Time-kill kinetics

Time-kill studies were conducted to assess bactericidal activity (60). Herbal monomers were diluted in Friis medium to final concentrations of 1 ×, 2 ×, and 4 × MIC. A mixture of 0.5 mL bacterial suspension (1 × 10⁶ CCU/mL) and 0.5 mL of drug solution was added to 4 mL of fresh medium. A drug-free control group (0.5 mL of bacterial suspension in 4.5 mL of medium) was included. Samples (100 µL) were collected every 24 h and quantified using the CCU method. Bacterial survival curves were plotted with time as the x-axis and the logarithmic viable count as the y-axis to evaluate bactericidal dynamics.

### Checkerboard assay for drug combinations

Synergistic activity of the herbal monomers and standard antimicrobials against Mhp was evaluated using a checkerboard microdilution assay (61). Each drug was serially diluted 2-fold in Friis medium across eight concentrations, starting at 4 × MIC. In 96-well plates, 50 µL of each diluted drug A was added along rows and drug B along columns, followed by 100 µL of bacterial suspension (1 × 10⁵ CCU/mL). Plates were incubated at 37°C, and growth was assessed every 12 h by monitoring color changes. The fractional inhibitory concentration index (FICI) was calculated as follows: FICI = (MIC_A combination/MIC_A alone) + (MIC_B combination/MIC_B alone). Interpretation: Synergy: FICI ≤ 0.5; Additive: 0.5 < FICI ≤ 1; Indifference: 1 < FICI ≤ 4; and Antagonism: FICI > 4 (62).

### Cytotoxicity assessment

Cytotoxicity of the herbal monomers on STEC, PK-15, and 3D4/21 cells was evaluated using the CCK-8 assay (63). Cells were trypsinized, counted, and seeded at 1 × 10⁵‒2 × 10⁵ cells/mL in 96-well plates (100 µL/well). After 24 h of incubation, cells were washed three times with PBS and treated with various concentrations of the herbal monomers (non-toxic range), each in 3–6 replicates. Control wells included a blank (no cells or drugs), a negative control (cells without drugs), and a positive control (10% Triton X-100). After 24 h of drug treatment, cells were washed, and 100 µL of 10% CCK-8 solution was added. After 1–4 h of incubation, absorbance was measured at 450 nm using a microplate reader. All experiments were performed in triplicate.

### Cell protection assay against ES-2 infection

Methods available in the literature were referenced and optimized as appropriate (64). The protective effects of the herbal monomers were evaluated at their maximum nontoxic concentrations. Cells were seeded in 96-well plates at 1 × 10⁴ cells/well and cultured overnight. When confluence reached 70%‒80%, they were infected with Mhp (1 × 10 CCU/mL) for 6 h. After three PBS washes, cells were treated with herbal compounds (diluted in 2% FBS DMEM) and incubated for 12, 24, and 48 h. Control groups included positive (10% Triton X-100), negative (untreated, infected), and blank controls. CCK-8 assays were performed at each time point to assess cell viability, with optical density measured at 450 nm.

### Pro-inflammatory cytokine expression analysis

To examine the anti-inflammatory effects of compounds, cells were seeded in 6-well plates at 2 × 10⁵ cells/mL (64). After reaching approximately 80% confluence, the cells were infected with Mhp (2 × 10 CCU/mL) for 6 h, washed with PBS, and treated with non-toxic concentrations of the compounds diluted in 2% FBS 1640 medium. After 24 h of incubation, cells were lysed in 1 mL of TRIzol reagent, and total RNA was extracted for cDNA synthesis following the manufacturer’s instructions. qPCR was conducted using an AceQ qPCR SYBR Green Master Mix on a CFX96 Real-Time PCR System (Bio-Rad) with the following conditions: 95°C for 10 s, followed by 40 cycles of 95°C for 10 s and 60°C for 30 s. Primer sequences are listed in Table S2. Gene expression was analyzed using the 2⁻ΔΔCT method (65).

### Animal infection model

This assay referred to the methods available in a previous study and optimized them appropriately (29). Twelve 21-day-old Chang Da binary cross-breeding piglets were confirmed to be negative for Mhp, and PRRSV antibodies were purchased from a local pig farm in Hubei Province, China. Only clinically healthy animals free of these pathogens, as confirmed by diagnostic testing, were used. All columns have been disinfected, uniformly treated, and randomly assigned. The piglets were randomly allocated to four groups (*n* = 3 per group): untreated control, FFC-treated, berberrubine-treated, and uninfected blank control groups. All piglets, except those in the blank control group, were inoculated intratracheally with Mhp ES-2 strain at a dose of 5 × 10⁸ CCU per pig. Six hours post-infection, the first dose of treatment was administered intramuscularly, followed by two additional doses at 72-h intervals (three doses in total). The treatment regimens for each group were as follows. Untreated control group: intramuscular injection of 0.1 mL/kg sterile saline; FFC treatment group: intramuscular injection of FFC at 20 mg/kg; berberrubine treatment group: intramuscular injection of berberrubine at 2.5 mg/kg; and blank control group: intramuscular injection of 0.1 mL/kg sterile saline without infection.

Body weight was measured on days 0 and 28 post-infection. ADG was calculated for each animal. Rectal temperature was recorded at regular intervals (prior to treatment and on days 0, 7, 8, 9, 14, 21, and 28 post-infection) to monitor fever progression in each group. On day 28 post-infection, all animals were euthanized. Pigs were humanely euthanized using a two-step method to ensure the rapid loss of consciousness followed by death. Animals were first sedated with an intramuscular injection of tiletamine-zolazepam (5 mg/kg body weight, Zoletil) combined with xylazine hydrochloride (2 mg/kg body weight) to induce deep sedation and minimize stress. Once the animals were fully sedated, euthanasia was performed via intravenous administration of pentobarbital sodium (150 mg/kg body weight, Euthasol). Administration was performed via the ear vein. Loss of consciousness was confirmed by the absence of corneal and palpebral reflexes, and death was confirmed by the cessation of heartbeat and respiratory movement. All procedures were carried out by trained personnel under the supervision of a licensed veterinarian to ensure animal welfare. Tissue samples were collected for further analysis. DNA was extracted from lung tissues using a commercial Mycoplasma Genomic DNA Extraction Kit following the manufacturer’s instructions. DNA samples were stored at −80°C in nuclease-free tubes for subsequent quantification of Mhp loads via qPCR. Additionally, total RNA was extracted from spleen, lymph node, and lung tissues to assess the expression of inflammatory cytokines using qPCR. Lung tissues were fixed in 4% paraformaldehyde and subjected to hematoxylin and eosin (H&E) staining for histopathological examination.

### Statistical analysis

The experimental data are expressed as mean ± standard deviation. Data were analyzed using a two-tailed unpaired *t*-test or one-way analysis of variance in GraphPad Prism (version 8.0). Statistical significance is expressed as follows: *P < 0.05, **P < 0.01, ***P < 0.001, ****P < 0.0001, ns, no significant difference.

## Data Availability

The data that support the findings of this study are openly available in Mendeley Data at 10.17632/xgmvtwxprk.1.

## SUPPLEMENTAL MATERIAL

Supplemental figures and tables spectrum.03254-25-s0001.docx Tables S1 to S2, and Figures S1 and S2.

ASM does not own the copyrights to Supplemental Material that may be linked to, or accessed through, an article. The authors have granted ASM a non-exclusive, world-wide license to publish the Supplemental Material files. Please contact the corresponding author directly for reuse.
