# Supplementary material for: In vitro and in vivo antimicrobial activity of protoberberine alkaloids as novel therapeutic candidates against Mycoplasma hyopneumoniae
Source: Microbiol Spectr. 2026 Jan 23;14(3):e03254-25. doi: 10.1128/spectrum.03254-25 (PMC12955479; doi:10.1128/spectrum.03254-25)
Supplement: Supplemental figures and tables — Tables S1 to S2, and Figures S1 and S2. [file spectrum.03254-25-s0001.docx]

Supplementary Materials

Table. S1.96 blank labels correspond to drug names.

| Annotation | Drugs |
| --- | --- |
| A | Epiberberine |
| B | Berberine |
| C | Phellodendrine Ketone |
| D | Berberrubine |
| E | Oxyberberine |
| F | Tetrahydroberberine |
| H | Ferulic Acid |
| I | Coptisine |
| J | Jatrorrhizine |
| 1 | Sennosides |
| 2 | Triptolide |
| 3 | Embelin |
| 4 | Hederacoside B |
| 5 | Flavopurpurin |
| 6 | 7, 8-Dihydroxyflavone |
| 7 | Berberis Gum |
| 8 | Punicalagin |
| 9 | Liquiritigenin |
| 10 | Baicalin |
| 11 | Siegesbeckia Herb |

Table S2. Sequence of primers in current study in qPCR

| Genes | Primer sequences（5’-3’） | Products size(bp) |
| --- | --- | --- |
| *P36*-F | GATTAGTGTCTCCAGTTATGAATATA | 242 |
| *P36*-R | GATCGGAAAATCCAGAAGCAT |  |
| GADPH-F | CCCCAACGTGTCGGTTGT | 65 |
| GADPH-R | CCTGCTTCACCACCTTCTTGA |  |
| IL-1β-F | GCCCATCATCCTTGAAACGTG | 110 |
| IL-1β-R | GGAGAGCCTTCAGCATGTGT |  |
| IL-6-F | CCACCGGTCTTGTGGAGTTT | 120 |
| IL-6-R | GTCTGGATTCTTTCCCTTTTGCC |  |
| TNF-α-F | GCCCTTCCACCAACGTTTTC | 87 |
| TNF-α-R | CAAGGGCTCTTGATGGCAGA |  |


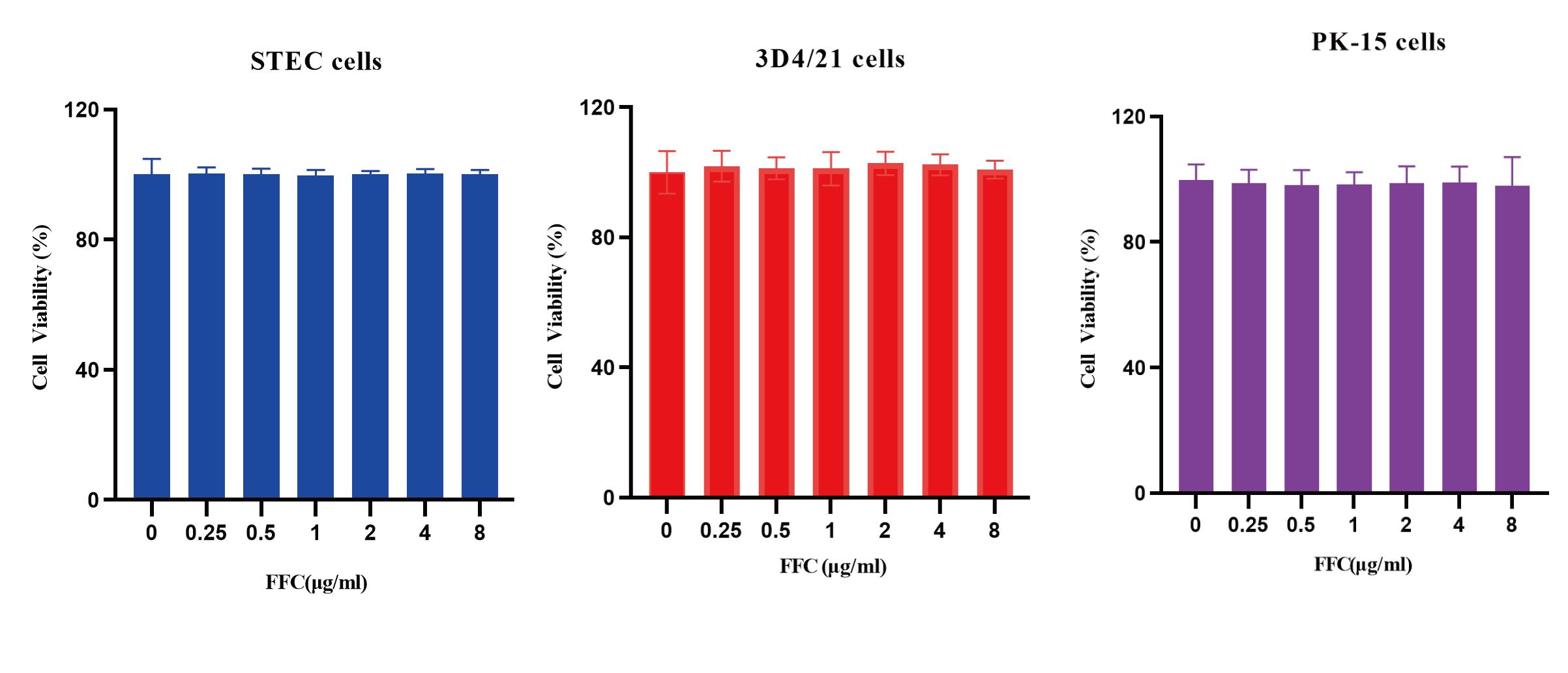


Figure. S1. Toxic effects of florfenicol on three cell lines. Data were mean values ± standard deviation of at least three biological replicates.


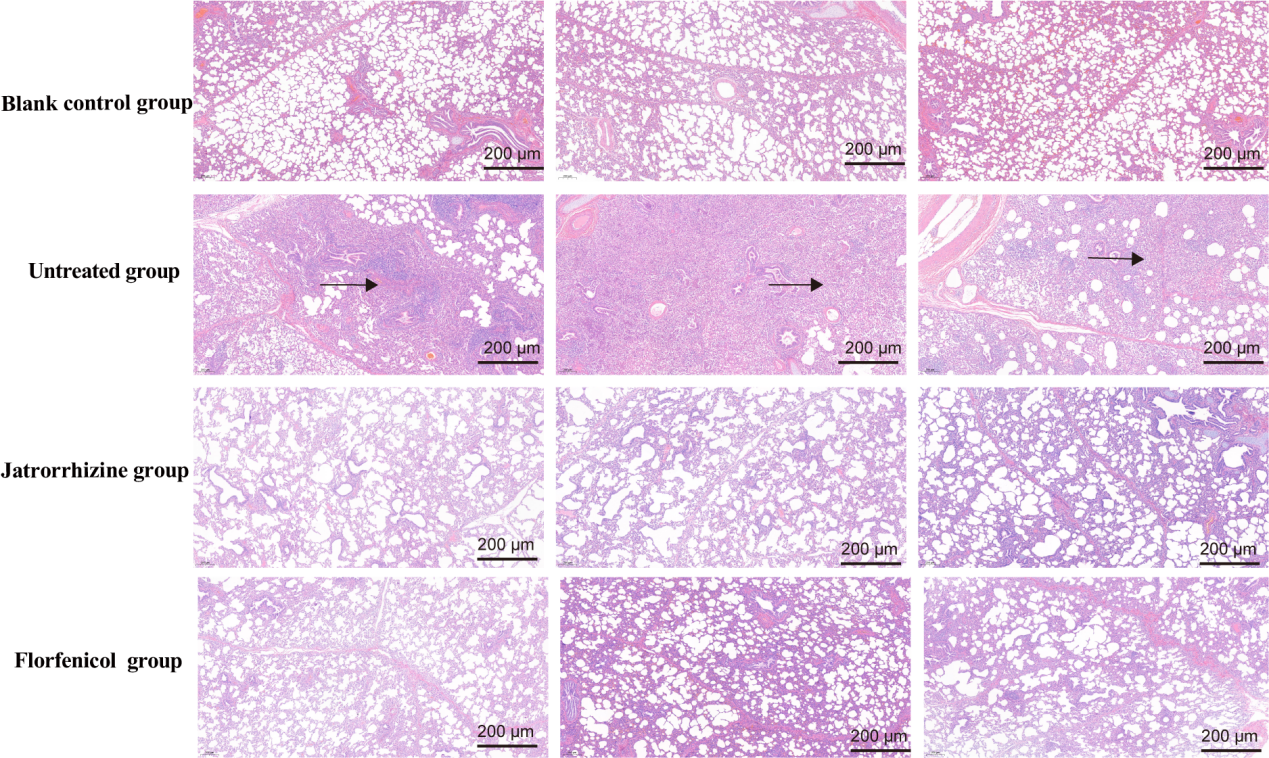


Figure. S2. Histopathological analysis of lung tissues from each Mhp-infected piglets after treatment with jatrorrhizine or florfenicol. Representative hematoxylin and eosin (H&E)-stained lung sections from four experimental groups: Blank control group (healthy piglets without infection), Untreated group (infected without treatment), Jatrorrhizine-treated group, and Florfenicol-treated group. Scale bar = 200 μm.
